# Regulatory Issues Surrounding Audit of Electronic Cigarette Charge Composition

**DOI:** 10.3389/fpsyt.2015.00133

**Published:** 2015-09-23

**Authors:** Mirjana Jovanovic, Mihajlo Jakovljevic

**Affiliations:** ^1^Department of Pharmacology and Toxicology, The Faculty of Medical Sciences, University of Kragujevac, Kragujevac, Serbia; ^2^Department of Psychiatry, The Faculty of Medical Sciences, University of Kragujevac, Kragujevac, Serbia

**Keywords:** tobacco, electronic cigarettes, nicotine, regulatory affairs, legislative framework, audit, control

## Clinical Case Triggering Attention to the Issue

A 29-year-old patient who was treated with buprenorphine for 2 years without recurrence attends regular psychiatric controls that include screening for psychoactive substances. During one of the visits, when he was accompanied by his parents, a test for psychoactive substances was positive for opiates. The patient denied that he used illegal substances, and his parents claimed so as well. During this period, the patient lived fairly in isolation, with the members of his family. The only new substance that he used was a new cartridge for e-cigarette. The cartridge was bought at the market in another place, at a pretty low price. Since the father used the same cartridge, he offered to take the test on PAS. The test was positive for opiates (father had never used psychoactive substances in his life). This inspired us to do the following experiment: we poured the content of the cartridge into a glass with water, and the test was again positive for opiates. In this case, neither the outcome of the test of our patient and/or his father nor naive experiment (with the help of immunochromatographic test for the simultaneous qualitative detection of drugs and their metabolites in the urine) or experimental method that is highly dubious was important, but the questions (i.e., more questions) that open after these clinical experiences.

The questions posed are: does any authority regularly control the composition of e-cigarettes, and if so, which one, under what circumstances, and whether it is regulated by law?

Let us start from the known premise: electronic cigarettes (e-cigarettes) are battery-powered devices that allow nicotine intake with chemicals that have different tastes by inhalation, and they are substitutes for smoking ordinary cigarettes. It is known that there are over 250 different brands of e-cigarettes currently on the market. It is estimated that the number of users of e-cigarettes in the world is rapidly growing. In Europe, there are about 7 million users of e-cigarettes. In France, there are about 1.5 million e-cigarette users, while, for example, in the UK, the number of e-cigarette users has tripled since 2012 (from 700,000 to over 2 million) (1).

The tobacco industry is investing a huge sum of money into the development of e-cigarettes, and according to the researchers from HSPH’s Center for Global Tobacco Control (CGTC), Department of Social and Behavioral Sciences, it is very important to identify the subpopulation that will probably use them more than others and determine the implications for public health. This research has shown that millions of people – including many young people and smokers who want to stop smoking – try e-cigarettes. This also points to the fact that the importance of determining the potential harm (or benefit) is being underestimated (2). Each e-cigarette contains the following components: batteries (mainly lithium ion) that can be automatic or manual; electronic atomizer spray (responsible for controlling the operation of the device and the release of nicotine vapor during inhalation); and tank in which the liquid for e-cigarette is poured (newer versions have atomizer and tank in one unit, and such a device is called clearomizer). The first e-cigarette appeared on the market in China in 2004, and since then, it is marketed as a healthier alternative to smoking. The biggest advantage of e-cigarette is, as stated in the advertisements, that the smoker inhales only a controlled dose of nicotine vapor that fulfills his need for smoking but does not inhale tar, carbon monoxide, remnants of metal, mercury, and many other harmful substances existing in each real cigarette. Also, according to advertising, “it protects and preserves the health of passive smokers, children also.”

## Are e-Cigarettes Safe?

E-cigarettes contain nicotine and other potentially dangerous substances. Except for nicotine, which is known to be highly addictive substance, other found chemicals, such as formaldehyde and acetaldehyde, are toxic and carcinogenic. In inhaled air from e-cigarette, nanoparticles of metal were found. Health consequences after consuming e-cigarettes are not known (3).

Users of e-cigarettes use cartridges with different concentrations of nicotine (and other substances), and so, potentially, they may be exposed to toxic concentrations of the same. The concentration of nicotine was ranked in the range from 0 to 34 mg/mL, but recent studies show a discrepancy between the indicated concentrations of nicotine on the bottle with filling and measured concentrations (4). E-cigarettes produce aerosol, which contains nicotine, and since that aerosol is heated (temperature depends on the design of the e-cigarette), it affects the aerosolization of the nicotine and its activity (5). Nicotine affects the peripheral nervous system and the central nervous system and represents the primary addictive ingredient with non-nicotine substances, such as anabasine, nornicotine, and acetaldehyde, which also affect the addiction to tobacco. FDA analysis showed the presence of anabasine in several types of e-cigarettes (6). In 20 different models of the e-cigarette, the presence of alkaloids similar to nicotine, such as nornicotine and anabasine, was found (7). Given that e-cigarettes are so different in design and content of nicotine, it makes it difficult to compare and assess the pharmacological properties of content, and therefore, the addictive and toxic potential.

## Do Regulatory Authorities Conduct Periodical Audit of Composition of e-Cigarette Cartridges?

Until recently, in most European countries, e-cigarettes were not treated either as a tobacco product or as a medical agent. This means that the only law that controls them is the national Consumer Protection Act that deals exclusively with the technical characteristics of products. Many contemporary laws on tobacco in the EU did not mention e-cigarettes as a tobacco product, and so they were not subject to stricter regulations. European Parliament in February 2014 approved that the products that have nicotine concentrations up to 20 mg/mL can be considered as tobacco products and that those with a higher concentration or used for therapeutic purposes can be considered a medicinal agent (8). However, this decision is left to the member countries themselves. This Directive insists on the health warning, mandatory information regarding the ingredients, and the side effects that package must contain.

Cartridges that can be refilled are allowed if their volume does not exceed 2 mL (or if at least three member countries estimate that they are potentially dangerous to health, they may be prohibited by the European Commission). In the UK, although there were indications that the e-cigarette will be declared a medical agent that is used for smokers who want to stop this habit, this has not happened still. E-cigarettes are treated as a “consumer product.” Restrictions relating to the marketing of e-cigarettes have not yet been introduced (9). The US Department of Health and Human Services (FDA) has proposed in April 2014 a set of regulatory rules relating to the control of e-cigarettes. Just e-cigarettes that are used for therapeutic purposes are currently regulated by the FDA (Center for Drug Evaluation and Research – CDER).

According to the FDA reports, voluntary reports of adverse effects of e-cigarettes, which include reports of consumers, medical professionals, and public, are regularly arriving. Adverse events that required even hospitalization described in these reports are pneumonia, congestive heart failure, disorientation, epileptic seizures, hypotension, and other health problems (10). E-cigarettes are manufactured in China and quality control is variable (11). Users can modify many products and even use other substances (e.g., marijuana) via e-cigarette.

## State-of-the-Art Clinical Research on e-Cigarettes

A relatively small number of clinical studies investigate the effect of e-cigarettes on health of people. Some of them show that e-cigarettes can “deliver” a similar amount of nicotine as traditional cigarettes (12, 13). Others show that the utilization of nicotine from e-cigarettes depends on the user experience and habits in use (7). Bahl et al. tested for cytotoxicity in 41 e-cigarettes produced by four different companies, using three types of cells: lung fibroblasts, embryonic stem cells (both of human origin), and neural stem cells of a mouse. Cytotoxicity varied among the products from highly toxic to non-toxic. Nicotine did not cause toxicity but other components did. It is important to point out that what was non-toxic to lung fibroblasts was extremely toxic to both types of stem cells (14). The research done by Schober and his associates measuring pollution in the room where three people smoked e-cigarette over a period of 2 h is interesting. The increase in the concentration of nicotine, 1,2-propanediol, aluminum, glycerin, and seven polycyclic aromatic hydrocarbons were classified as probably carcinogenic by the International Agency for research on Cancer (15). Propylene glycol and glycerin are the main ingredients of cigarettes. Exposure to propylene glycol can lead to irritation of the eyes and lungs, and repeated inhalation leads to effects on the CNS, behavior, and results in damage to the spleen (16). The conclusion is that a very small number of studies investigate the direct health effects, but some suggest that aerosols from e-cigarettes have biological effect. Long-term biological effects are still unknown, given that e-cigarettes are a relatively new invention.

Let us go back to the beginning of the story: it is obvious that we do not know the composition of e-cigarettes, even when we think it is known and controlled by the competent regulatory authorities. The health effects of “known” components of aerosols emitted by e-cigarettes are unknown. There are not enough research/clinical studies on the topic of the impact/risk to human health.

Increasingly, these electronic devices are used for the enjoyment of other PAS. Marijuana is mostly used in liquid form or in the form of wax. It is perfect for users since there is no characteristic odor that occurs when smoking marijuana. This way, unimpeded by police or similar services, illegal drugs can be used or smuggled. This is certainly a new reason to think about.

Concern regarding the quality control and health outcomes is justified. It will be necessary to evolve legal framework to regulate the production and circulation of e-cigarettes and determine their actual effect on health. Anyway, one of the first steps is regulation that will allow finally that e-cigarettes come under the scrutiny of the professional public for its initial market access stage. Thus, in the upcoming years, full clinical potential as well as room to avoid key adverse events would become better known to the consumers and addictologists as well.

## Conflict of Interest Statement

The authors declare that the research was conducted in the absence of any commercial or financial relationships that could be construed as a potential conflict of interest.
